# Associations of Serum Folate and Vitamin B_12_ Levels With Cardiovascular Disease Mortality Among Patients With Type 2 Diabetes

**DOI:** 10.1001/jamanetworkopen.2021.46124

**Published:** 2022-01-31

**Authors:** Yujie Liu, Tingting Geng, Zhenzhen Wan, Qi Lu, Xuena Zhang, Zixin Qiu, Lin Li, Kai Zhu, Liegang Liu, An Pan, Gang Liu

**Affiliations:** 1 Department of Nutrition and Food Hygiene, Hubei Key Laboratory of Food Nutrition and Safety, Ministry of Education Key Lab of Environment and Health, and State Key Laboratory of Environmental Health (Incubating), School of Public Health, Tongji Medical College, Huazhong University of Science and Technology, Wuhan, China; 2 Department of Epidemiology and Biostatistics, School of Public Health, Tongji Medical College, Huazhong University of Science and Technology, Wuhan, China

## Abstract

**Importance:**

The associations of serum folate and vitamin B_12_ levels with cardiovascular outcomes among patients with type 2 diabetes (T2D) remain unclear.

**Objective:**

To investigate the associations of serum folate and vitamin B_12_ levels with risk of cardiovascular disease (CVD) mortality among individuals with T2D.

**Design, Setting, and Participants:**

This prospective cohort study included 8067 patients with T2D who participated in the National Health and Nutrition Examination Survey (NHANES) from 1999 through 2014 and NHANES III (1988-1994). American Diabetes Association criteria were used to define T2D. Data were analyzed between October 1, 2020, and April 1, 2021.

**Exposures:**

Serum folate and vitamin B_12_ levels.

**Main Outcomes and Measures:**

Multivariable Cox proportional hazards regression models were used to compute hazard ratios and 95% CIs for the associations of serum folate and vitamin B_12_ levels with risks of CVD and all-cause mortality. Two multivariable models were constructed. Restricted cubic spline analyses were used to examine the nonlinear association of serum folate levels and vitamin B_12_ levels with CVD mortality, and nonlinearity was assessed using the likelihood ratio test.

**Results:**

This cohort study included data from 7700 participants in the folate analysis (mean [SE] age, 57.8 [0.3] years; 3882 men [weighted, 50.5%]; median serum folate level, 12.1 ng/mL [IQR, 7.1-19.5 ng/mL]) and 4860 participants for the vitamin B_12_ analysis (mean [SE] age, 57.8 [0.3] years; 2390 men [weighted, 50.7%]; median serum vitamin B_12_ level, 506.1 pg/mL [IQR, 369.1-703.5 pg/mL]). During 72 031 person-years of follow-up, 799 CVD deaths were documented for the folate analysis, and during 43 855 person-years of follow-up, 467 CVD deaths were reported for the vitamin B_12_ analysis. Nonlinear associations were observed for serum levels of folate (*P* = .04 for nonlinearity) and vitamin B_12_ (*P* = .04 for nonlinearity) with risk of CVD mortality among patients with T2D. Compared with participants in the second quartile of serum folate levels (7.1-12.1 ng/mL), the hazard ratios for CVD mortality were 1.43 (95% CI, 1.04-1.98) for participants in the lowest serum folate level quartile (<7.1 ng/mL) and 1.03 (95% CI, 0.74-1.44) for participants in the highest quartile (≥19.5 ng/mL). In addition, compared with participants in the second quartile of serum vitamin B_12_ levels (369.1-506.0 pg/mL), the hazard ratios for CVD mortality were 1.74 (95% CI, 1.20-2.52) for participants in the lowest quartile (<369.1 pg/mL) and 2.32 (95% CI, 1.60-3.35) for participants in the highest quartile (≥703.5 pg/mL). Similar patterns of association were observed for all-cause mortality (nonlinearity: *P* = .01 for folate and *P* = .02 for vitamin B_12_).

**Conclusions and Relevance:**

This cohort study found that both low and high serum levels of vitamin B_12_ as well as low serum levels of folate were significantly associated with higher risk of CVD mortality among individuals with T2D.

## Key Points

**Question:**

Are serum folate and vitamin B_12_ levels associated with cardiovascular disease (CVD) mortality among patients with type 2 diabetes?

**Findings:**

In this cohort study of 8067 patients with type 2 diabetes, results suggested nonlinear associations of serum folate and vitamin B_12_ levels with risk of CVD mortality. Both low and high levels of serum vitamin B_12_ were associated with a higher risk of CVD mortality, whereas low levels of serum folate were associated with a higher risk of CVD mortality.

**Meaning:**

These findings suggest that maintaining moderate levels of serum folate and vitamin B_12_ may decrease the risk of CVD death among patients with type 2 diabetes.

## Teaser

This cohort study uses data from a nationally representative sample of US adults to assess whether serum folate or vitamin B_12_ levels are associated with the risk of cardiovascular disease mortality among individuals with type 2 diabetes.

## Introduction

Folate (vitamin B_9_) and cobalamin (vitamin B_12_), involved in 1-carbon metabolism, are essential nutrients for nucleotide and amino acid biosynthesis.^1,2^ Folate and vitamin B_12_ deficiency have been associated with anemia^3^ and cognitive function in general populations,^4,5,6,7^ with additional risk of neural defects,^8,9^ cardiovascular disease (CVD),^10,11,12^ and diabetes^13^ for folate deficiency.

Among patients with type 2 diabetes (T2D), vitamin B_12_ deficiency is common owing to medication interactions (eg, metformin) or malnutrition.^14,15,16^ In addition, previous studies have shown that circulating folate concentrations are significantly lower in patients with T2D compared with healthy individuals.^17^ Hence, it is important to investigate the association of folate and vitamin B_12_ status with long-term health outcomes among patients with T2D.

Previous epidemiological studies have shown conflicting results between folate concentrations and mortality among people with diabetes, with some studies reporting a positive dose-response association^18,19^ and other studies observing no association.^20^ However, those previous studies have relatively small sample sizes ranging from 526 to 689 and insufficient adjustment for essential confounders (eg, duration of diabetes, medication use, and glycemic control). In addition, to our knowledge, the evidence suggesting an association between vitamin B_12_ levels and mortality among patients with T2D is scarce. To address these research gaps, we prospectively examine the associations of serum folate levels and vitamin B_12_ levels with risks of CVD and all-cause mortality in a nationally representative sample of US adults with T2D.

## Methods

### Study Population

The National Health and Nutrition Examination Survey (NHANES) is a periodic, cross-sectional sampling survey conducted by the National Center for Health Statistics of the Centers for Disease Control and Prevention and is a nationally representative sample of the noninstitutionalized US civilian population. Participants provided information on demographic and socioeconomic characteristics and health-related behaviors and health conditions using standardized questionnaires. The questionnaires were administered and collected at study recruitment by trained interviewers. Physical measurements and laboratory tests were also administered by trained medical professionals in mobile examination centers. No measurements were repeated. The details of the sampling methods and analytic guidelines have been published elsewhere.^21^ This study followed the Strengthening the Reporting of Observational Studies in Epidemiology (STROBE) reporting guideline for cohort studies. The protocols of the NHANES study were approved by the Institutional Review Board of the National Center of Health Statistics, and all participants provided informed written consent at enrollment. No one received compensation or was offered any incentive for participating in this study.

In this cohort study, we included participants with diabetes (≥20 years of age) from NHANES III (1988-1994) and from 8 cycles of NHANES from 1999 through 2014. Data on vitamin B_12_ were not available from NHANES III (1988-1991) and NHANES 2007 through 2010. We defined T2D based on participants’ meeting 1 of the American Diabetes Association criteria: (1) self-reported physician diagnosis of diabetes; (2) receipt of oral glucose-lowering medicines or insulin; and (3) fasting plasma glucose level of at least 126 mg/dL, 75-g oral glucose tolerance test of at least 200 mg/dL (to convert glucose to millimoles per liter, multiply by 0.0555), or hemoglobin A_1c_ (HbA_1c_) level of at least 6.5% (48 mmol/mol) (to convert HbA_1c_ percentage of total hemoglobin to a proportion of total hemoglobin, multiply by 0.01). In total, 9289 participants met the diagnostic criteria for diabetes. After excluding participants who were pregnant (n = 22), had prevalent cancer (n = 1193), or were lost to follow-up (n = 7), 8067 participants with T2D were included. Of them, data for 7700 participants were included in the final folate analyses, and data for 4860 participants were included in the final vitamin B_12_ analyses.

### Laboratory Measurements

Blood samples were processed, frozen at −20 °C, and sent to the National Center for Environmental Health for testing. A detailed description of the laboratory methods can be found on the NHANES website.^21^

Because previous evidence has indicated a stronger association between serum folate and homocysteine levels rather than between red blood cell folate and homocysteine levels,^22,23^ we used serum folate levels in the present study. Total serum folate levels were measured using different methods for different years that NHANES was conducted: 1999 through 2000, affinity/high-performance liquid chromatography (HPLC) with electrochemical (coulometric) detection; NHANES III (1988-1994) and 2001 through 2006, Bio-Rad Laboratories Quantaphase II radioimmunoassay kit for folate and vitamin B_12_; 2007 through 2010, microbiologic assay; and 2011 through 2014, isotope-dilution HPLC coupled with tandem mass spectrometry. Because it has been previously suggested that, of these methods, the microbiologic assay is most accurate, serum folate values from 1999 through 2006 were converted to equivalent values from 2007 through 2014 by using fractional polynomial regression.^24,25,26^

Serum vitamin B_12_ levels were determined using the Bio-Rad Laboratories Quantaphase II radioimmunoassay for folate and vitamin B_12_ in NHANES III and in 1999 through 2006 and using the fully automated Roche electrochemiluminescence immunoassay in 2011 through 2014. As suggested, vitamin B_12_ data from NHANES III (1991-1994) and from 1999 through 2006 were converted using the Deming regression model so that the values were comparable to those obtained from 2011 through 2014 by using the Roche assay.^24,27^ The coefficient of variation was controlled within 10% for serum folate and within 5% for serum vitamin B_12_.

### Assessment of Covariates

Race and ethnicity were assessed as basic demographic variables and categorized based on self-report in the interview. Body mass index (BMI) was calculated as weight in kilograms divided by height in meters squared and was categorized as lower than 25, 25 to 30, or 30 or higher.^28^ Participants were classified as being a nondrinker, low to moderate drinker, or heavy drinker according to the self-reported average number of alcoholic drinks consumed per day. A moderate drinker was defined as having fewer than 2 drinks per day for men and fewer than 1 drink per day for women; a heavy drinker was defined as 2 or more drinks per day for men and 1 or more drinks per day for women. Being physically active was defined as participating in moderate-intensity or vigorous sports, fitness programs, or recreational activities more than 10 minutes per week, otherwise participants were considered inactive if they did not exercise more than 10 minutes per week. Data on physician-diagnosed history of hypertension, hypercholesterolemia, and CVD were self-reported. Information on medications and dietary supplements taken during the past 30 days was collected by trained professionals through matching the products provided by the participants with the drug and dietary supplement database. In addition, levels of homocysteine, triglyceride, total cholesterol, high-density lipoprotein cholesterol, and low-density lipoprotein cholesterol were measured at recruitment.

### Ascertainment of Mortality

Data for deaths were obtained by linking the cohort database with the National Death Index through December 31, 2015. All-cause mortality was defined as any reason for death. We defined CVD mortality using the *International Statistical Classification of Diseases and Related Health Problems, Tenth Revision* codes I00 to I09, I11, I13, I20 to I51, and I60 to I69.

### Statistical Analysis

Given the complex sampling design of NHANES, all analyses in the present study incorporated sample weights, clustering, and stratification. Each participant’s person-years were calculated from the date of recruitment to the date of death or the end of follow-up (December 31, 2015), whichever occurred first. Multivariable Cox proportional hazards regression models were used to compute hazard ratios (HRs) and 95% CIs for the associations of serum folate and vitamin B_12_ levels with risks of CVD and all-cause mortality. Schoenfeld residuals were used to test the proportional hazards assumption, and no violation was observed. Two multivariable models were constructed. In model 1, we adjusted for age (continuous or years), sex (male or female), and race and ethnicity (self-reported Mexican American, non-Hispanic Black, non-Hispanic White, or other, which included other Hispanic, other non-Hispanic race, and non-Hispanic multiracial). In model 2, we additionally adjusted for educational level (<high school, high school or equivalent, or ≥college), family ratio of income to poverty (<1.0, 1.0-3.0, or >3.0), BMI (<25.0, 25.0-29.9, or ≥30.0), smoking status (never, ever, or current), alcohol consumption (nondrinker, low to moderate, or heavy), moderate to vigorous physical activity (inactive or active), healthy eating index (continuous), duration of diabetes (<3 years, 3-10 years, or >10 years), diabetes medication use (none, only oral medication, insulin, or others), HbA_1c_ level (<7.0% or ≥7.0%), and history of hypertension or hypercholesterolemia. Variables with missing values were imputed using the multiple imputation method.

Restricted cubic spline analysis with 4 knots (5th, 35th, 65th, and 95th percentiles) was used to examine the nonlinear association of serum folate levels and vitamin B_12_ levels with CVD mortality (25th percentile as reference) within the values between the first and 95th percentile to minimize the influence of potential outliers. Nonlinearity was tested using the likelihood ratio test. The associations of the quartiles of serum folate and vitamin B_12_ levels with mortality were examined using the second quartile as the reference group based on the results of restricted cubic spline analyses.

We further stratified the analyses by age (<60 or ≥60 years), sex (male or female), race and ethnicity (non-White or other), smoking status (never, ever, or current), alcohol consumption (nondrinker or drinker), physically active (inactive or active), BMI (<30 or ≥30), HbA_1c_ (<7% or ≥7%), hypertension (yes or no), hypercholesterolemia (yes or no), and diabetes duration (<10 or ≥10 years). The *P* values for the production terms between serum folate and vitamin B_12_ levels and the stratified factors were used to estimate the significance of interactions. The interaction between serum folate levels and vitamin B_12_ levels was also tested.

We also conducted a series of sensitivity analyses. (1) To minimize the potential reverse causation bias, we excluded participants who died within 2 years of follow-up. (2) We repeated the main analyses according to quintiles of serum folate and vitamin B_12_ levels. (3) Serum folate and vitamin B_12_ levels were mutually adjusted. (4) Participants with a history of CVD were further excluded from the main analyses. (5) Vitamin B_12_ supplementation and folate supplementation were additionally adjusted. (6) Dietary vitamin B_12_ and folate intake were additionally adjusted. (7) To investigate a potential role of inflammation, blood lipid levels, liver and kidney indices, or homocysteine levels with any of the observed associations, we further adjusted for C-reactive protein (CRP) levels, lipid profile (including triglycerides, low-density lipoprotein cholesterol, and high-density lipoprotein cholesterol), an indicator of kidney function (estimated glomerular filtration rate), and indicators of liver function (levels of aspartate aminotransferase, alanine transaminase, gamma-glutamyl transpeptidase, lactate dehydrogenase, and circulating homocysteine [only available in subsamples]). (8) We performed the main analysis by categorizing quartile 4 of the vitamin B_12_ level as 2 groups (<950.0 pg/mL and ≥950.0 pg/mL; to convert vitamin B_12_ levels to picomoles per liter, multiply by 0.7378) because some clinical recommendations have suggested that the standard reference range of serum vitamin B_12_ for healthy adults is between 160 and 950 pg/mL (118 and 701 pmol/L).^29^ In addition, because serum folate levels and serum vitamin B_12_ levels were not normally distributed, associations at baseline were tested by using unadjusted Spearman correlation coefficients. We also performed a restricted cubic spline analysis that included all values as a sensitivity analysis.

All analyses were performed using SAS, version 9.4 (SAS Institute Inc), and a 2-sided *P* < .05 was set as the threshold for statistical significance. Data were analyzed between October 1, 2020, and April 1, 2021.

## Results

For the analysis of serum folate levels (7700 adults; mean [SE] age, 57.8 (0.3) years; 3882 men [weighted, 50.5%] and 3818 women [weighted, 49.5%]; median folate level, 12.1 ng/mL [IQR, 7.1-19.5 ng/mL] [to convert folate levels to nanomoles per liter, multiply by 2.266]), we identified 2749 all-cause deaths and 799 CVD deaths during 72 031 person-years of follow-up. For the analysis of serum vitamin B_12_ levels (4860 adults; mean (SE), age 57.8 (0.3) years; 2390 men [weighted, 50.7%] and 2470 women [weighted, 49.3%]; median vitamin B_12_ level, 506.1 pg/mL [IQR, 369.1-703.5 pg/mL]), we identified 1650 all-cause deaths and 467 CVD deaths during 43 855 person-years of follow-up. The baseline characteristics of participants by quartile of serum folate and vitamin B_12_ levels are given in Table 1. Compared with 1921 participants in the second quartile of folate levels, 1929 participants in the lowest quartile were more likely to be younger (mean [SE] age, 54.5 [0.5] vs 55.2 [0.5] years), women (1002 [49.0%] vs 909 [47.5%]), non-Hispanic Black (671 [22.5%] vs 550 [18.9%]), less educated (some college or more, 357 [32.0%] vs 566 [39.3%]), current smokers (472 [29.3%] vs 393 [22.3%]), and heavy drinkers (144 [8.2%] vs 94 [5.1%]) and tended to have lower prevalence rates of hypertension (1026 [53.7%] vs 1088 [56.4%]) and hypercholesterolemia (546 [38.0%] vs 830 [47.5%]). Compared with 1216 participants in the second quartile of vitamin B_12_ levels, 1213 participants in the lowest quartile were more likely to be older (mean [SE] age, 58.8 [0.5] vs 56.9 [0.6] years), men (men (658 [53.6%] vs 626 [52.1%]), non-Hispanic White (523 [68.0%] vs 421 [63.2%]), never smokers (592 [49.9%] vs 572 [47.2%]), and physically inactive (761 [59.6%] vs 733 [53.6%]) and tended to have a longer duration of diabetes (>10 years, 316 [25.8%] vs 279 [21.8%]) and higher prevalence rates of hypertension (722 [62.1%] vs 691 [57.1%]) and hypercholesterolemia (577 [55.7%] vs 523 [51.5%]); 1215 participants in the highest quartile compared with 1216 in the second quartile were likely to be older (mean [SE] age, 58.8 [0.5] vs 56.9 [0.6] years), women (669 [53.8%] vs 590 [47.9%]), non-Hispanic Black (401 [18.8%] vs 239 [12.4%]), and physically inactive (755 [56.0%] vs 733 [53.6%]) and tended to have a longer duration of diabetes (>10 years, 359 [30.2%] vs 279 [21.8%]).

**Table 1.** Baseline Characteristics of Participants With Diabetes by Serum Folate and Vitamin B_12_ Levels in NHANES III (1988-1994) and NHANES 1999 Through 2014^a^

Characteristic	Participants, No. (%)									
	Serum folate level, ng/mL					Serum vitamin B_12_ level, pg/mL				
	Total	Quartile 1 (<7.1)	Quartile 2 (7.1-12.1)	Quartile 3 (12.2-19.4)	Quartile 4 (≥19.5)	Total	Quartile 1 (<369.1)	Quartile 2 (369.1-506.0)	Quartile 3 (506.1-703.4)	Quartile 4 (≥703.5)
Participants, No.	7700	1929	1921	1925	1925	4860	1213	1216	1216	1215
Age, mean (SE), y	57.8 (0.3)	54.5 (0.5)	55.2 (0.5)	57.2 (0.4)	62.1 (0.4)	57.8 (0.3)	58.8 (0.5)	56.9 (0.6)	56.7 (0.6)	58.8 (0.5)
HEI, mean (SE)	54.3 (0.3)	54.1 (0.7)	52.0 (0.5)	53.5 (0.5)	57.1 (0.5)	53.5 (0.5)	52.7 (0.7)	52.9 (0.7)	53.0 (0.7)	55.7 (0.7)
Sex										
Female	3818 (49.5)	1002 (49.0)	909 (47.5)	926 (46.4)	1045 (54.3)	2470 (49.3)	555 (46.4)	590 (47.9)	656 (49.7)	669 (53.8)
Male	3882(50.5)	927 (51.0)	1012 (52.5)	999 (53.6)	880 (45.7)	2390 (50.7)	658 (53.6)	626 (52.1)	560 (50.3)	546 (46.2)
Race and ethnicity										
Mexican American	1976 (9.4)	630 (8.5)	504 (10.0)	477 (10.7)	365 (8.2)	1234 (9.4)	285 (7.2)	351 (10.4)	321 (10.4)	277 (9.9)
Non-Hispanic										
Black	2059 (15.5)	671 (22.5)	550 (18.9)	464 (14.4)	374 (10.5)	1349 (15.7)	256 (11.6)	293 (12.4)	399 (20.8)	401 (18.8)
White	2681 (61.3)	532 (58.8)	642 (57.5)	674 (60.5)	833 (66.4)	1625 (60.4)	523 (68.0)	421 (63.2)	334 (52.4)	347 (56.3)
Other^b^	984 (13.7)	96 (10.3)	225 (13.6)	310 (14.3)	353 (14.8)	652 (14.6)	149 (13.2)	151 (14.0)	162 (16.4)	190 (15.1)
Educational level										
<High school	3296 (29.3)	946 (33.5)	842 (33.1)	803 (28.0)	705 (25.5)	2059 (29.2)	501 (27.9)	509 (27.9)	545 (32.7)	504 (28.6)
High school	2073 (28.2)	626 (34.5)	513 (27.6)	452 (26.3)	482 (27.5)	1279 (28.2)	321 (27.9)	322 (28.0)	322 (29.7)	314 (27.4)
Some college or more	2331 (42.5)	357 (32.0)	566 (39.3)	670 (45.8)	738 (46.9)	1522 (42.6)	391 (44.3)	385 (44.2)	349 (37.6)	397 (44.1)
BMI										
<25.0	1218 (14.2)	315 (14.6)	288 (13.2)	293 (13.2)	322 (15.7)	747 (13.9)	163 (10.9)	170 (12.1)	182 (14.4)	232 (18.7)
25.0-29.9	2457 (28.9)	628 (29.3)	577 (25.8)	616 (29.1)	636 (31.0)	1555 (29.2)	382 (28.8)	359 (23.5)	394 (32.3)	420 (32.7)
≥30.0	3843 (57.0)	949 (56.2)	1011 (61.0)	970 (57.7)	913 (53.3)	2436 (57.0)	634 (60.3)	659 (64.5)	610 (53.4)	533 (48.6)
Family income to poverty ratio										
<1.0	1801 (17.8)	539 (20.5)	470 (20.1)	426 (17.9)	366 (14.5)	1189 (18.8)	288 (17.3)	296 (17.4)	314 (22.1)	291 (19.0)
1.0-3.0	3268 (42.5)	803 (46.1)	804 (42.0)	812 (40.5)	849 (43.1)	2042 (42.0)	501 (42.0)	523 (43.0)	505 (40.1)	513 (42.7)
>3.0	1881 (39.7)	363 (33.3)	461 (37.9)	511 (41.6)	546 (42.5)	1208 (39.2)	323 (40.6)	302 (39.5)	289 (37.9)	294 (38.4)
Smoking status										
Never	3721 (48.0)	848 (39.8)	892 (49.1)	971 (48.8)	1010 (50.3)	2398 (48.3)	592 (49.9)	572 (47.2)	623 (48.8)	611 (47.1)
Ever	2582 (33.3)	609 (30.9)	633 (28.7)	620 (31.8)	720 (39.5)	1607 (33.1)	404 (30.6)	419 (34.1)	374 (31.4)	410 (36.5)
Current	1389 (18.8)	472 (29.3)	393 (22.3)	329 (19.4)	195 (10.3)	848 (18.6)	215 (19.5)	225 (18.7)	214 (19.8)	194 (16.4)
Alcohol consumption										
None	3443 (38.7)	1137 (48.3)	821 (38.4)	731 (34.7)	754 (38.0)	2142 (38.4)	502 (39.0)	551 (38.6)	534 (36.7)	555 (38.9)
Low to moderate	3347 (55.3)	553 (43.6)	900 (56.5)	950 (59.4)	944 (56.4)	2182 (56.2)	569 (55.8)	537 (54.6)	551 (58.3)	525 (56.4)
Heavy	419 (6.0)	144 (8.2)	94 (5.1)	96 (5.9)	85 (5.6)	240 (5.4)	59 (5.2)	68 (6.7)	55 (5.0)	58 (4.7)
Physical activity										
Inactive	4974 (59.4)	1339 (61.6)	1231 (61.5)	1191 (57.7)	1213 (58.5)	2994 (56.7)	761 (59.6)	733 (53.6)	745 (57.5)	755 (56.0)
Active	2725 (40.6)	590 (38.4)	690 (38.5)	734 (42.4)	711 (41.5)	1865 (43.3)	452 (40.4)	483 (46.4)	470 (42.5)	460 (44.0)
Duration of diabetes, y										
<3	3951 (53.0)	1153 (63.7)	976 (55.0)	959 (50.8)	863 (48.5)	2414 (51.3)	628 (52.1)	631 (56.8)	616 (52.9)	539 (42.9)
3-10	1563 (22.2)	300 (17.0)	422 (23.1)	430 (24.5)	411 (21.9)	1030 (23.5)	234 (22.1)	258 (21.5)	251 (23.9)	287 (26.9)
>10	1933 (24.7)	383 (19.3)	461 (21.9)	493 (24.7)	596 (29.6)	1253 (25.2)	316 (25.8)	279 (21.8)	299 (23.1)	359 (30.2)
HbA_1c_, %										
<7.0	4358 (58.3)	1138 (62.6)	1032 (55.0)	1025 (55.7)	1163 (61.3)	2664 (56.8)	725 (62.9)	672 (55.8)	637 (53.2)	630 (54.5)
≥7.0	3320 (41.7)	787 (37.4)	884 (45.0)	893 (44.3)	756 (38.7)	2188 (43.2)	488 (37.1)	543 (44.2)	576 (46.8)	581 (45.6)
Self-reported disease										
Hypertension	4517 (58.8)	1026 (53.7)	1088 (56.4)	1150 (58.3)	1253 (63.7)	2836 (58.6)	722 (62.1)	691 (57.1)	705 (57.2)	718 (57.7)
Hypercholesterolemia	3381 (50.8)	546 (38.0)	830 (47.5)	949 (53.1)	1056 (57.4)	2218 (52.4)	577 (55.7)	523 (51.5)	548 (50.3)	570 (51.9)

Abbreviations: BMI, body mass index (calculated as weight in kilograms divided by height in meters squared); HbA_1c_, glycated hemoglobin A_1c_; HEI, Healthy Eating Index; NHANES, National Health and Nutrition Examination Survey.

SI conversion factors: To convert serum levels of vitamin B_12_ to picomoles per liter, multiply by 0.7378; folate levels to nanomoles per liter, multiply by 2.266; and HbA_1c_ levels to a proportion of total hemoglobin, multiply by 0.01.

^a^ All estimates accounted for complex survey designs, and all percentages were weighted.

^b^ Categorized based on self-report in the NHANES interview.

### Serum Folate and Mortality

A nonlinear association was observed between serum folate level and CVD mortality (*P* = .04 for nonlinearity) (Figure, A). After multivariable adjustment, compared with the reference group (the second quartile), the HRs of CVD mortality were 1.43 (95% CI, 1.04-1.98) in the first quartile, 1.17 (95% CI, 0.88-1.57) in the third quartile, and 1.03 (95% CI, 0.74-1.44) in the fourth quartile (Table 2). Similarly, compared with the reference group (quartile 2), the HRs of all-cause mortality were 1.17 (95% CI, 1.01-1.37) for the first quartile, 1.02 (95% CI, 0.84-1.24) for the third quartile, and 1.07 (95% CI, 0.91-1.28) for the fourth quartile (*P* = .01 for nonlinearity) (Table 2).

**Figure.**
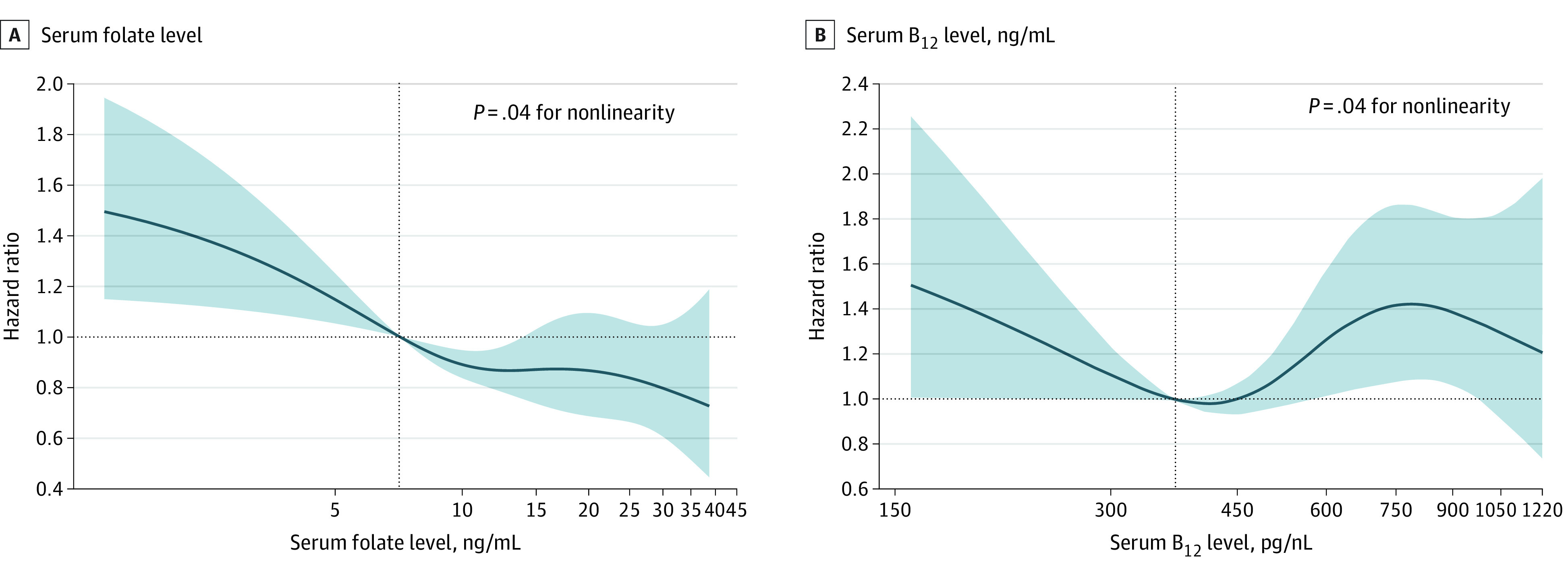
Association of Serum Folate and Vitamin B_12_ Levels With Cardiovascular Disease Mortality Among Adults With Diabetes in the National Health and Nutrition Examination Survey III (NHANES; 1988-1994) and NHANES 1999 Through 2014 Hazard ratios (solid lines) and 95% CIs (shaded areas) were adjusted for age (continuous), sex (male or female), race and ethnicity (self-reported Mexican American, non-Hispanic Black, non-Hispanic White, or other), body mass index (calculated as weight in kilograms divided by height in meters squared; <25.0, 25.0-29.9, or ≥30.0), educational level (<high school, high school or equivalent, or ≥college), family income level (lower, normal, or higher), smoking status (never, ever, or current), alcohol consumption (none, low to moderate, or heavy), physical activity (inactive or active), Healthy Eating Index (continuous), duration of diabetes (<3, 3-10, or >10 years), diabetes medication use (none, oral glucose-lowering medication, only insulin, or oral glucose-lowering medication and insulin), glycated hemoglobin A_1c_ (<7% or ≥7%), self-reported hypertension (yes or no), and self-reported hypercholesterolemia (yes or no). Vertical dotted lines indicate the 25th percentile. To convert serum levels of vitamin B_12_ to picomoles per liter, multiply by 0.7378; folate to nanomoles per liter, multiply by 2.266.

**Table 2.** Hazard ratios of CVD and All-Cause Mortality by Folate and Vitamin B_12_ Serum Levels Among Adults With Diabetes in NHANES III (1988-1994) and NHANES 1999 Through 2014

Model	Hazard ratio (95% CI)							
	Folate serum level, ng/mL				Vitamin B_12_ serum level, pg/mL			
	Quartile 1 (<7.1)	Quartile 2 (7.1-12.1)	Quartile 3 (12.2-19.4)	Quartile 4 (≥19.5)	Quartile 1 (<369.1)	Quartile 2 (369.1-506.0)	Quartile 3 (506.1-703.4)	Quartile 4 (≥703.5)
**CVD mortality**								
Deaths, No./total No.	345/1929	180/1921	159/1925	115/1925	138/1213	88/1216	117/1216	124/1215
Model 1^a^	1.42 (1.00-2.01)	1 [Reference]	1.09 (0.81-1.47)	0.96 (0.69-1.34)	1.78 (1.15-2.77)	1 [Reference]	1.65 (1.07-2.54)	2.49 (1.70-3.64)
Model 2^b^	1.43 (1.04-1.98)	1 [Reference]	1.17 (0.88-1.57)	1.03 (0.74-1.44)	1.74 (1.20-2.52)	1 [Reference]	1.79 (1.19-2.71)	2.32 (1.60-3.35)
**All-cause mortality**								
Deaths, No./total No.	1085/1929	680/1921	503/1925	481/1925	452/1213	393/1216	401/1216	404/1215
Model 1^a^	1.19 (1.00-1.42)	1 [Reference]	0.96 (0.80-1.16)	0.99 (0.84-1.16)	1.10 (0.92-1.32)	1 [Reference]	1.33 (1.08-1.62)	1.34 (1.09-1.64)
Model 2^b^	1.17 (1.01-1.37)	1 [Reference]	1.02 (0.84-1.24)	1.07 (0.91-1.28)	1.11 (0.94-1.32)	1 [Reference]	1.37 (1.10-1.70)	1.29 (1.06-1.58)

Abbreviations: CVD, cardiovascular disease; NHANES, National Health and Nutrition Examination Survey.

SI conversion factors: To convert serum levels of vitamin B_12_ to picomoles per liter, multiply by 0.7378; folate to nanomoles per liter, multiply by 2.266; and glycated hemoglobin A_1c_ levels to a proportion of total hemoglobin, multiply by 0.01.

^a^ Adjusted for age (continuous), sex (male or female), and race (non-Hispanic White, non-Hispanic Black, Mexican American, or other).

^b^ Further adjusted for body mass index (calculated as weight in kilograms divided by height in meters squared; <25.0, 25.0-29.9, or ≥30.0), educational level (<high school, high school or equivalent, or ≥college), family income level (lower, normal, or higher), smoking status (never, ever, or current), alcohol consumption (none, low to moderate, or heavy), physical activity (inactive or active), Healthy Eating Index (continuous), duration of diabetes (<3, 3-10, or >10 years), diabetes medication use (none, only oral glucose-lowering medication, only insulin, or oral medication and insulin), glycated hemoglobin A_1c_ (<7% or ≥7%), self-reported hypertension (yes or no), and self-reported hypercholesterolemia (yes or no).

### Serum Vitamin B_12_ and Mortality

A nonlinear association was found between serum vitamin B_12_ level and CVD mortality (*P* = .04 for nonlinearity) (Figure, B). After multivariable adjustment, both lower and higher levels of vitamin B_12_ were associated with higher risk of CVD mortality. Compared with the reference group (the second quartile), the HRs of CVD mortality were 1.74 (95% CI, 1.20-2.52) in the first quartile, 1.79 (95% CI, 1.19-2.71) in the third quartile, and 2.32 (95% CI, 1.60-3.35) in the fourth quartile (Table 2). In addition, higher levels of serum vitamin B_12_ were associated with higher risk of all-cause mortality. Compared with the reference group (the second quartile), the HRs of all-cause mortality were 1.11 (95% CI, 0.94-1.32) in the first quartile, 1.37 (95% CI, 1.10-1.70) in the third quartile, and 1.29 (95% CI, 1.06-1.58) in the fourth quartile (*P* = .02 for nonlinearity) (Table 2).

### Stratified and Sensitivity Analyses

We found a significant interaction between serum folate and HbA_1c_ levels with the risk of CVD mortality (*P* = .02 for interaction). For the subgroup with HbA_1c_ levels less than 7.0% compared with the reference group (the second quartile), the HR of CVD mortality was 1.18 (95% CI, 0.62-2.24) in the first quartile, and in the subgroup with HbA_1c_ levels 7.0% or higher compared with the reference group, the HR of CVD mortality was 2.65 (95% CI, 1.46-4.81) in the first quartile. However, no significant interactions were found between serum folate level, vitamin B_12_ level, or any other strata variables with the risk of CVD or all-cause mortality after correcting for multiple testing (Table 3; eTables 1 and 2 in the Supplement), and no significant interaction was found between serum folate and vitamin B_12_ levels among individuals with T2D (χ^2^ = 6.32, *P* = .71 for all-cause mortality and χ^2^ = 13.33, *P* = .15 for CVD mortality, both for interaction).

**Table 3.** Associations of Folate and Vitamin B_12_ Serum Levels With CVD Mortality in Various Subgroups Among Adults With Diabetes in NHANES III (1988-1994) and NHANES 2001 Through 2014

Characteristic	Hazard ratio (95% CIs) by quartile^a^				*P* value for interaction
	Quartile 1	Quartile 2	Quartile 3	Quartile 4	
Folate serum level, ng/mL	<7.1	7.1-12.1	12.2-19.4	≥19.5	
Age, y					
<60	1.98 (0.85-4.62)	1 [Reference]	1.19 (0.54-2.66)	0.97 (0.34-2.72)	.76
≥60	1.22 (0.91-1.65)	1 [Reference]	1.12 (0.83-1.51)	1.20 (0.87-1.66)	
Sex					
Female	1.14 (0.77-1.69)	1 [Reference]	0.77 (0.50-1.17)	0.77 (0.48-1.25)	.82
Male	1.78 (1.17-2.71)	1 [Reference]	1.55 (1.05-2.29)	1.32 (0.83-2.08)	
Race and ethnicity					
Non-Hispanic White	1.36 (0.90-2.05)	1 [Reference]	0.98 (0.68-1.42)	0.93 (0.65-1.34)	.35
Other^b^	1.97 (1.28-3.01)	1 [Reference]	1.79 (1.19-2.70)	1.40 (0.82-2.40)	
BMI					
<30	1.73 (1.03-2.88)	1 [Reference]	1.21 (0.76-1.93)	0.72 (0.42-1.23)	.96
≥30	1.85 (0.89-3.85)	1 [Reference]	1.59 (0.93-2.71)	1.87 (1.04-3.37)	
Smoking status					
Never	1.37 (0.84-2.24)	1 [Reference]	1.34 (0.81-2.22)	1.07 (0.66-1.73)	.21
Ever or current	1.56 (1.09-2.23)	1 [Reference]	1.08 (0.72-1.60)	1.02 (0.63-1.67)	
Alcohol consumption					
Nondrinker	1.37 (0.98-1.92)	1 [Reference]	1.11 (0.78-1.59)	0.83 (0.53-1.30)	.82
Drinker	1.57 (0.90-2.76)	1 [Reference]	1.29 (0.84-1.98)	1.28 (0.81-2.04)	
Diabetes duration, y					
<10	1.76 (0.93-3.35)	1 [Reference]	1.29 (0.80-2.09)	0.83 (0.48-1.44)	.17
≥10	1.51 (0.69-3.31)	1 [Reference]	1.43 (0.83-2.47)	1.50 (0.75-3.00)	
Physical activity					
Inactive	1.53 (0.94-2.49)	1 [Reference]	1.27 (0.79-2.04)	1.11 (0.66-1.86)	.30
Active	2.41 (0.89-6.54)	1 [Reference]	1.79 (0.98-3.29)	1.29 (0.54-3.08)	
HbA_1c_, %					
<7.0	1.18 (0.62-2.24)	1 [Reference]	1.05 (0.63-1.75)	0.91 (0.61-1.35)	.02
≥7.0	2.65 (1.46-4.81)	1 [Reference]	1.80 (1.12-2.91)	1.46 (0.66-3.23)	
Hypertension					
Yes	1.84 (1.06-3.20)	1 [Reference]	1.37 (0.85-2.19)	1.47 (0.91-2.36)	.33
No	1.97 (1.03-3.78)	1 [Reference]	1.49 (0.74-3.03)	0.74 (0.30-1.79)	
Hypercholesterolemia					
Yes	2.55 (1.28-5.08)	1 [Reference]	1.73 (1.02-2.95)	1.56 (0.82-2.97)	.16
No	1.27 (0.69-2.33)	1 [Reference]	1.19 (0.70-2.03)	0.85 (0.47-1.55)	
Vitamin B_12_ serum level, pg/mL	<369.1	369.1-506.0	506.1-703.4	≥703.5	
Age, y					
<60	1.13 (0.74-1.73)	1 [Reference]	1.78 (1.13-2.81)	1.71 (1.05-2.77)	.48
≥60	1.07 (0.91-1.25)	1 [Reference]	1.10 (0.86-1.40)	1.14 (0.92-1.41)	
Sex					
Female	1.09 (0.83-1.44)	1 [Reference]	1.42 (1.06-1.89)	1.23 (0.88-1.70)	.75
Male	1.11 (0.86-1.43)	1 [Reference]	1.30 (0.94-1.79)	1.36 (1.01-1.82)	
Race and ethnicity					
Non-Hispanic White	1.01 (0.83-1.22)	1 [Reference]	1.43 (1.08-1.88)	1.27 (0.94-1.71)	.97
Other^b^	1.48 (1.09-2.01)	1 [Reference]	1.28 (0.95-1.71)	1.40 (0.99-1.98)	
BMI					
<30	0.87 (0.66-1.13)	1 [Reference]	1.11 (0.84-1.48)	1.19 (0.89-1.59)	.53
≥30	1.33 (1.01-1.76)	1 [Reference]	1.59 (1.18-2.15)	1.29 (0.93-1.80)	
Smoking status					
Never	1.08 (0.79-1.48)	1 [Reference]	1.44 (1.05-1.97)	1.34 (0.95-1.89)	.85
Ever or current	1.15 (0.91-1.45)	1 [Reference]	1.36 (1.01-1.83)	1.25 (0.95-1.66)	
Alcohol consumption					
Nondrinker	1.15 (0.91-1.46)	1 [Reference]	1.40 (1.06-1.85)	1.19 (0.91-1.56)	.17
Drinker	1.06 (0.80-1.41)	1 [Reference]	1.33 (0.97-1.81)	1.29 (0.92-1.80)	
Diabetes duration, y					
<10	1.18 (0.92-1.50)	1 [Reference]	1.46 (1.13-1.90)	1.54 (1.15-2.07)	.58
≥10	1.07 (0.80-1.44)	1 [Reference]	1.29 (0.92-1.81)	1.09 (0.80-1.48)	
Physical activity					
Inactive	1.24 (1.00-1.54)	1 [Reference]	1.51 (1.20-1.90)	1.42 (1.14-1.77)	.97
Active	0.86 (0.62-1.20)	1 [Reference]	1.19 (0.83-1.72)	1.18 (0.81-1.71)	
HbA_1c_, %					
<7.0	1.97 (1.12-3.46)	1 [Reference]	1.67 (1.00-2.79)	2.93 (1.65-5.21)	.94
≥7.0	1.53 (0.81-2.91)	1 [Reference]	1.69 (0.90-3.16)	1.79 (1.06-3.04)	
Hypertension					
Yes	1.47 (0.92-2.33)	1 [Reference]	1.52 (0.93-2.51)	1.94 (1.19-3.16)	.80
No	2.55 (1.15-5.67)	1 [Reference]	2.24 (1.05-4.80)	3.73 (1.73-8.08)	
Hypercholesterolemia					
Yes	1.05 (0.82-1.35)	1 [Reference]	1.38 (1.00-1.91)	0.90 (0.68-1.19)	.29
No	1.15 (0.88-1.50)	1 [Reference]	1.37 (1.06-1.78)	1.85 (1.39-2.46)	

Abbreviations: BMI, body mass index (calculated as weight in kilograms divided by height in meters squared); CVD, cardiovascular disease; HbA_1c_, glycated hemoglobin A_1c_; NHANES, National Health and Nutrition Examination Survey.

SI conversion factors: To convert serum levels of vitamin B_12_ to picomoles per liter, multiply by 0.7378; folate to nanomoles per liter, multiply by 2.266; and HbA_1c_ levels to a proportion of total hemoglobin, multiply by 0.01.

^a^ Adjusted for age (continuous), sex (male or female), race and ethnicity (Mexican American, non-Hispanic Black, non-Hispanic White, or other), BMI (<25.0, 25.0-29.9, or ≥30.0), educational level (<high school, high school or equivalent, or ≥college), family income level (lower, normal, or higher), smoking status (never, ever, or current), alcohol consumption (none, low to moderate, or heavy), physical activity (inactive or active), Healthy Eating Index (continuous), duration of diabetes (<3, 3-10, or >10 years), diabetes medication use (none, only oral glucose-lowering medication, only insulin, or oral medication and insulin), HbA_1c_ (<7% or ≥7%), self-reported hypertension (yes or no), and self-reported hypercholesterolemia (yes or no). The strata variable was not included when stratifying by itself.

^b^ Categorized based on self-report in the NHANES interview.

The results were generally robust in sensitivity analyses when excluding the participants who died within 2 years of follow-up (eTable 3 in the Supplement), repeating the main analyses by quintiles of serum folate and vitamin B_12_ levels (eTable 4 in the Supplement), mutually adjusting for serum folate and vitamin B_12_ levels (model 2; eTable 5 in the Supplement), further excluding participants who had a history of CVD at baseline (model 3; eTable 5 in the Supplement), further adjusting for supplemental and dietary intake of vitamin B_12_ and folate (models 8 and 9; eTable 5 in the Supplement), or performing the main analyses by dividing quartile 4 of vitamin B_12_ levels into 2 groups (eTable 6 in the Supplement). The associations did not materially change when further adjusting for CRP levels, lipid levels, or liver function–related or kidney function–related indicators (models 4, 5, 7, and 8; eTable 5 in the Supplement). When circulating homocysteine was further adjusted, the association was attenuated for levels of serum folate but not vitamin B_12_ (model 6; eTable 5 in the Supplement). The restricted cubic spline did not materially change when all values were included (eFigure in the Supplement). Vitamin B_12_ levels remained significantly associated with BMI and levels of HbA_1c_, CRP, triglycerides, high-density lipoprotein cholesterol, and homocysteine; folate levels remained significantly associated with age and CRP, total cholesterol, triglycerides, low-density lipoprotein cholesterol, high-density lipoprotein cholesterol, and homocysteine levels (eTable 7 in the Supplement).

## Discussion

In this large, prospective cohort study of US adults with diabetes, we found significant nonlinear associations between serum folate and vitamin B_12_ levels and CVD mortality. A low serum folate level (<7.1 ng/mL) was associated with a higher risk of CVD mortality. In addition, both low (<369.1 pg/mL) and high (≥506.1 pg/mL) serum levels of vitamin B_12_ were associated with a higher risk of CVD mortality. A variety of stratified analyses and sensitivity analyses indicated the robustness of our findings.

The association between serum folate level and mortality has been examined in different populations with mixed findings. In a general population in NHANES 1999 through 2010, Peng et al^24^ found that low folate levels (in the first quartile) were significantly associated with higher risk of all-cause and CVD mortality in comparison with participants in the second folate level quartile. Among participants with rheumatoid arthritis in NHANES III (1988-1994) and 2011, circulating folate levels were inversely associated with risk of all-cause mortality and CVD mortality,^25^ whereas a U-shaped association between serum folate level and CVD mortality was observed among adults with hypertension.^26^ However, among adults with T2D, the evidence regarding folate status and long-term health outcomes is limited and mixed. One previous study of 689 US adults found that serum folate level was not associated with the risk of CVD mortality among participants with diabetes,^20^ whereas 2 other studies using data from NHANES 1991 through 1994 showed that serum and red blood cell folate levels were positively associated with mortality risk.^18,19^ Those inconsistent findings could be partially due to small sample size (eg, only including 532 individuals with diabetes in the latter 2 studies). Moreover, some important confounding factors, such as duration of diabetes, medications for diabetes, and glycemic control, are not considered in those studies. In the present study with a larger sample size, we found nonlinear associations between serum folate level and CVD and all-cause mortality among 7700 US adults with diabetes, and low (<7.1 ng/mL) but not high serum folate levels were significantly associated with higher risk of CVD and all-cause mortality after adjusting for potential confounders.

For vitamin B_12_ level, the association between its status and mortality has been relatively underexamined. In a general population, data from NHANES 1999 through 2006 and 2011 through 2014 showed that low levels of serum vitamin B_12_ were associated with a moderate increase in all-cause mortality, and both low and higher serum levels of vitamin B_12_ were associated with a small but significant increase in CVD mortality.^27^ Furthermore, the Newcastle 85+ study, which included 752 individuals 85 years of age or older, found that higher levels of plasma vitamin B_12_ were associated with higher risks of all-cause mortality and CVD mortality among women.^30^ However, among patients with diabetes, who often had high prevalence rates of vitamin B_12_ deficiency due to medication interactions (eg, metformin) or malnutrition,^14,15,16^ evidence regarding vitamin B_12_ status and long-term health outcomes is scarce. To our knowledge, only 1 Indian study has examined the association of circulating vitamin B_12_ levels with mortality among patients with diabetes (n = 396).^31^ That study showed that higher levels of serum vitamin B_12_ were associated with a higher risk of all-cause mortality. That study was limited by small sample size and insufficient adjustment of important confounders (ie, lifestyle factors and use of diabetes medication). Our study, with a larger sample size and full adjustment of potential confounders, found a nonlinear association between levels of serum vitamin B_12_ and CVD mortality (ie, both low [<369.1 pg/mL] and high serum vitamin B_12_ level [≥703.5 pg/mL] were associated with a higher risk of CVD mortality among adults with diabetes). In addition, higher levels of serum vitamin B_12_ (≥703.5 pg/mL) were associated with a higher risk of all-cause mortality among individuals with T2D.

The potential mechanisms underlying the association between low folate levels and mortality risk may be partially explained by homocysteine metabolism.^2^ Folate deficiency may lead to the accumulation of homocysteine, which has been associated with increased risk of stroke, CVD, dementia, and pregnancy complications.^1,32,33^ In the present study, the association between low levels of folate and risk of CVD mortality was not significant when serum homocysteine was further adjusted, suggesting that the observed association may be partially mediated via circulating homocysteine. In addition, previous intervention studies have shown that folate and vitamin B_12_ supplementation may reduce the risk of mortality^34^ and stroke^35^ by lowering circulating homocysteine levels. Our data showed that higher serum vitamin B_12_ levels were associated with a higher risk of CVD mortality independent of homocysteine levels. Increased vitamin B_12_ levels may be a result of decreased uptake by peripheral tissues or increased ingestion or therapeutic administration through 1 or more of the following mechanisms: elevated plasma levels of transcobalamin I/III, increased hepatic cytolysis, decreased vitamin B_12_ clearance by the liver, and decreased production of transcobalamin II.^36,37^ Elevated levels of serum vitamin B_12_ may also reflect functional vitamin B_12_ deficiency in the peripheral tissue or impaired liver or kidney function.^36,38^ Mechanistic studies are warranted to clarify the roles of serum folate and vitamin B_12_ levels in the long-term health of individuals with T2D.

### Strengths and Limitations

Our study has some strengths. To our knowledge, the present study is the largest investigation of the associations of serum folate and vitamin B_12_ levels with CVD and all-cause mortality among individuals with diabetes, with consideration of a multitude of potential confounding factors. In addition, the present analysis is based on a nationally representative sample of US adults with diabetes, which facilitates the generalization of the findings.

Our study also has some limitations. First, the circulating folate and vitamin B_12_ levels were based on a single serum measurement, which may not accurately reflect the long-term status. Second, covariates collected at baseline may change over time, which may attenuate the true association of serum folate and vitamin B_12_ levels with mortality. Third, the severity of diabetes could not be meticulously controlled for in the present analysis owing to a lack of information, although we adjusted for duration of diabetes, diabetic medications, and HbA_1c_ levels. Fourth, because we categorized the serum levels of folate and vitamin B_12_ based on quartiles of the study population, our results may not be comparable to other studies using different cut points. In addition, residual or unknown confounding cannot be entirely excluded. Fifth, owing to the nature of the observational study design, our findings cannot be used for inference of causality.

## Conclusions

Among a nationally representative sample of US adults with T2D, we found nonlinear associations of serum folate and vitamin B_12_ levels with CVD mortality. Low levels of serum folate were associated with a higher risk of CVD mortality, and both low and high levels of serum vitamin B_12_ were associated with a higher risk of CVD mortality. Our study results indicate a potential beneficial role of maintaining moderate levels of serum folate and vitamin B_12_ in decreasing the risk of CVD death among adults with T2D.

## Notes

Supplement. **eTable 1.** Associations Between Serum Folate With All-Cause Mortality in Various Subgroups Among Patients With T2D in NHANES III and NHANES 1999-2014 **eTable 2.** Associations Between Serum B_12_ With All-Cause Mortality in Various Subgroups Among Patients With T2D in NHANES III and NHANES 1999-2014 **eTable 3.** HRs (95% CIs) of CVD Mortality According to Serum Folate and Vitamin B_12_ Levels After Excluding T2D Patients With Less Than 2 Years of Follow-up **eTable 4.** HR (95% CIs) of CVD Mortality According to Quintiles of Serum Folate and Vitamin B_12_ Concentrations Among Patients With T2D **eTable 5.** HRs (95% CIs) of CVD Mortality According to Serum Folate and Vitamin B_12_ Levels Among T2D Patients With Further Adjustment of Several Biomarkers and Dietary Factors **eTable 6.** HRs (95% CIs) of CVD Mortality According to Serum Vitamin B_12_ Levels Among Patients With T2D in NHANES III and NHANES 1999-2014 **eTable 7.** Spearman Correlation of Baseline Serum Folate and B_12_ With Age, BMI, HbA1c, CRP, Triglycerides, LDL, HDL, and Homocysteine Among Patients With T2D **eFigure.** Association of Serum Folate (A) and Vitamin B_12_ (B) With CVD Mortality Among Patients With T2D
