# Errors in Figure and Table

**DOI:** 10.1001/jamanetworkopen.2022.4936

**Published:** 2022-03-08

In the Original Investigation titled “Associations of Serum Folate and Vitamin B_12_ Levels With Cardiovascular Disease Mortality Among Patients With Type 2 Diabetes,”^1^ published January 31, 2022, there were errors in panel B of the Figure; the unit of measure for serum B_12_ should be picograms per milliliter. There was an error in the title of Table 3; the years of the National Health and Nutrition Examination Survey should be from 1999 through 2014. This article has been corrected.^1^
