# Errors in Abstract, Results, and Table 1

**DOI:** 10.1001/jamanetworkopen.2023.19477

**Published:** 2023-06-05

In the Original Investigation titled “Associations of Serum Folate and Vitamin B_12_ Levels With Cardiovascular Disease Mortality Among Patients With Type 2 Diabetes,”^1^ published January 31, 2022, there were errors in the abstract, Results, and Table 1 regarding the numbers and weighted percentages of men and women in this cohort study. Of the 7700 participants, 3882 (weighted, 50.5%) were women, and 3818 (weighted, 49.5%) were men. This article has been corrected.^1^
