# Supplementary material for: Associations of Serum Folate and Vitamin B12 Levels With Cardiovascular Disease Mortality Among Patients With Type 2 Diabetes
Source: JAMA Netw Open. 2022 Jan 31;5(1):e2146124. doi: 10.1001/jamanetworkopen.2021.46124 (PMC8804919; doi:10.1001/jamanetworkopen.2021.46124)
Supplement: Supplement. — eTable 1. Associations Between Serum Folate With All-Cause Mortality in Various Subgroups Among Patients With T2D in NHANES III and NHANES 1999-2014 eTable 2. Associations Between Serum B12 With All-Cause Mortality in Various Subgroups Among Patients With T2D in NHANES III and NHANES 1999-2014 eTable 3. HRs (95% CIs) of CVD Mortality According to Serum Folate and Vitamin B12 Levels After Excluding T2D Patients With Less Than 2 Years of Follow-up eTable 4. HR (95% CIs) of CVD Mortality According to Quintiles of Serum Folate and Vitamin B12 Concentrations Among Patients With T2D eTable 5. HRs (95% CIs) of CVD Mortality According to Serum Folate and Vitamin B12 Levels Among T2D Patients With Further Adjustment of Several Biomarkers and Dietary Factors eTable 6. HRs (95% CIs) of CVD Mortality According to Serum Vitamin B12 Levels Among Patients With T2D in NHANES III and NHANES 1999-2014 eTable 7. Spearman Correlation of Baseline Serum Folate and B12 With Age, BMI, HbA1c, CRP, Triglycerides, LDL, HDL, and Homocysteine Among Patients With T2D eFigure. Association of Serum Folate (A) and Vitamin B12 (B) With CVD Mortality Among Patients With T2D [file jamanetwopen-e2146124-s001.pdf]

Supplementary Online Content

Liu Y, Geng T, Wan Z, et al. Associations of serum folate and vitamin B_{12} levels with cardiovascular disease mortality among patients with type 2 diabetes. *JAMA Netw Open*. 2022;5(1):e2146124. doi:10.1001/jamanetworkopen.2021.46124

eTable 1. Associations Between Serum Folate With All-Cause Mortality in Various Subgroups Among Patients With T2D in NHANES III and NHANES 1999-2014
eTable 2. Associations Between Serum B_{12} With All-Cause Mortality in Various Subgroups Among Patients With T2D in NHANES III and NHANES 1999-2014
eTable 3. HRs (95% CIs) of CVD Mortality According to Serum Folate and Vitamin B_{12} Levels After Excluding T2D Patients With Less Than 2 Years of Follow-up
eTable 4. HR (95% CIs) of CVD Mortality According to Quintiles of Serum Folate and Vitamin B_{12} Concentrations Among Patients With T2D
eTable 5. HRs (95% CIs) of CVD Mortality According to Serum Folate and Vitamin B_{12} Levels Among T2D Patients With Further Adjustment of Several Biomarkers and Dietary Factors
eTable 6. HRs (95% CIs) of CVD Mortality According to Serum Vitamin B_{12} Levels Among Patients With T2D in NHANES III and NHANES 1999-2014
eTable 7. Spearman Correlation of Baseline Serum Folate and B_{12} With Age, BMI, HbA1c, CRP, Triglycerides, LDL, HDL, and Homocysteine Among Patients With T2D
eFigure. Association of Serum Folate (A) and Vitamin B_{12} (B) With CVD Mortality Among Patients With T2D

This supplementary material has been provided by the authors to give readers additional information about their work.

© 2022 Liu Y et al. *JAMA Network Open*.

eTable 1. Associations Between Serum Folate With All-Cause Mortality in Various Subgroups Among Patients With T2D in NHANES III and NHANES 1999-2014

	Quartiles of serum folate, nmol/L				
	**Q1: <16.2**	**Q2: 16.2-27.4**	**Q3: 27.5-44.0**	**Q4: ≥44.1**	***P*** **interaction**
**Age, years**					.85
<60	1.00 (0.74, 1.35)	1.00 (ref.)	0.87 (0.61, 1.24)	1.09 (0.69, 1.73)	
≥60	1.16 (0.97, 1.38)	1.00 (ref.)	1.06 (0.86, 1.30)	1.28 (1.04, 1.57)	
**Sex**					.91
Female	1.07 (0.88, 1.30)	1.00 (ref.)	0.88 (0.67, 1.17)	1.12 (0.90, 1.40)	
Male	1.26 (1.00, 1.58)	1.00 (ref.)	1.10 (0.88, 1.38)	1.00 (0.77, 1.29)	
**Race**					.83
White	1.19 (0.98, 1.46)	1.00 (ref.)	1.08 (0.84, 1.39)	1.07 (0.86, 1.34)	
Other	1.21 (0.96, 1.53)	1.00 (ref.)	0.89 (0.69, 1.14)	1.15 (0.88, 1.49)	
**BMI, kg/m^2**					.02
<30	1.31 (0.96, 1.81)	1.00 (ref.)	1.39 (1.05, 1.86)	1.27 (0.96, 1.67)	
≥30	1.36 (0.99, 1.86)	1.00 (ref.)	0.98 (0.73, 1.31)	1.25 (0.93, 1.68)	
**Smoking status**					.10
Never	1.02 (0.81, 1.30)	1.00 (ref.)	1.03 (0.77, 1.37)	1.23 (0.97, 1.57)	
Ever or current	1.25 (1.03, 1.52)	1.00 (ref.)	0.99 (0.79, 1.23)	0.94 (0.75, 1.18)	
**Drinking status**					.18
Non-drinkers	1.03 (0.86, 1.25)	1.00 (ref.)	0.97 (0.75, 1.26)	1.11 (0.88, 1.39)	
Drinkers	1.35 (1.06, 1.71)	1.00 (ref.)	1.06 (0.84, 1.33)	1.04 (0.83, 1.31)	
**Duration, years**					.07
<10	1.28 (0.98, 1.69)	1.00 (ref.)	1.118 (0.81, 1.54)	1.02 (0.76, 1.36)	
≥10	1.17 (0.79, 1.72)	1.00 (ref.)	1.104 (0.77, 1.59)	1.43 (1.00, 2.03)	
**Physical activity**					.04
Inactive	1.18 (0.91, 1.54)	1.00 (ref.)	1.06 (0.81, 1.38)	1.15 (0.86, 1.54)	
Active	1.48 (0.97, 2.26)	1.00 (ref.)	1.29 (0.96, 1.73)	1.29 (0.90, 1.85)	
**HbA1c**					.40
<7.0%	1.36 (1.00, 1.85)	1.00 (ref.)	1.16 (0.80, 1.68)	1.18 (0.87, 1.62)	
≥7.0%	1.21 (0.87, 1.68)	1.00 (ref.)	1.07 (0.83, 1.38)	1.18 (0.82, 1.69)	
**Hypertension**					.27
Yes	1.43 (1.08, 1.89)	1.00 (ref.)	1.20 (0.90, 1.60)	1.29 (1.02, 1.64)	
No	1.04 (0.72, 1.50)	1.00 (ref.)	0.97 (0.66, 1.41)	1.10 (0.77, 1.56)	
**Hypercholesterolemia**					.11
Yes	1.21 (0.86, 1.70)	1.00 (ref.)	1.14 (0.85, 1.54)	1.20 (0.86, 1.67)	
No	1.27 (0.97, 1.67)	1.00 (ref.)	1.13 (0.81, 1.59)	1.24 (0.91, 1.68)	

HRs (95% CIs) were adjusted for age (continuous), sex (male, or female), race/ethnicity (non-Hispanic white, non-Hispanic black, Mexican American, or other), BMI (<25.0, 25.0-29.9, or ≥30.0 kg/m^2), education level (less than high school, high school or equivalent, or college or above), family income level (lower, normal, higher), smoking status (never smoker, ever smoker, or current smoker), drinking status (never drinker, ever drinker, or current drinker), physical activity (inactive, or active), HEI (continuous), duration of diabetes (<3, 3-10, or >10 years), diabetes medication use (none, only diabetes pills, only insulin, or diabetes pill and insulin), HbA1c (<7%, ≥7%), self-reported hypertension (yes or no), self-reported hypercholesterolemia (yes or no). The strata variable was not included when stratifying by itself.

© 2022 Liu Y et al. *JAMA Network Open*.

eTable 2. Associations Between Serum B_{12} With All-Cause Mortality in Various Subgroups Among Patients With T2D in NHANES III and NHANES 1999-2014

	Quartiles of serum vitamin B_{12}, pmol/L				
	**Q1: <272.3**	**Q2: 272.3-373.3**	**Q3: 373.4-519.0**	**Q4: ≥519.1**	***P* interaction**
**Age, years**					.48
<60	1.13 (0.74, 1.73)	1.00 (ref.)	1.78 (1.13, 2.81)	1.71 (1.05, 2.77)	
≥60	1.07 (0.91, 1.25)	1.00 (ref.)	1.10 (0.86, 1.40)	1.14 (0.92, 1.41)	
**Sex**					.75
Female	1.09 (0.83, 1.44)	1.00 (ref.)	1.42 (1.06, 1.89)	1.23 (0.88, 1.70)	
Male	1.11 (0.86, 1.43)	1.00 (ref.)	1.30 (0.94, 1.79)	1.36 (1.01, 1.82)	
**Race**					.97
White	1.01 (0.83, 1.22)	1.00 (ref.)	1.43 (1.08, 1.88)	1.27 (0.94, 1.71)	
Other	1.48 (1.09, 2.01)	1.00 (ref.)	1.28 (0.95, 1.71)	1.40 (0.99, 1.98)	
**BMI, kg/m^2**					.53
<30	0.87 (0.66, 1.13)	1.00 (ref.)	1.11 (0.84, 1.48)	1.19 (0.89, 1.59)	
≥30	1.33 (1.01, 1.76)	1.00 (ref.)	1.59 (1.18, 2.15)	1.29 (0.93, 1.80)	
**Smoking status**					.85
Never	1.08 (0.79, 1.48)	1.00 (ref.)	1.44 (1.05, 1.97)	1.34 (0.95, 1.89)	
Ever or current	1.15 (0.91, 1.45)	1.00 (ref.)	1.36 (1.01, 1.83)	1.25 (0.95, 1.66)	
**Drinking status**					.17
Non-drinkers	1.15 (0.91, 1.46)	1.00 (ref.)	1.40 (1.06, 1.85)	1.19 (0.91, 1.56)	
Drinkers	1.06 (0.80, 1.41)	1.00 (ref.)	1.33 (0.97, 1.81)	1.29 (0.92, 1.80)	
**Duration, years**					.58
<10	1.18 (0.92, 1.50)	1.00 (ref.)	1.46 (1.13, 1.90)	1.54 (1.15, 2.07)	
≥10	1.07 (0.80, 1.44)	1.00 (ref.)	1.29 (0.92, 1.81)	1.09 (0.80, 1.48)	
**Physical activity**					.97
Inactive	1.24 (1.00, 1.54)	1.00 (ref.)	1.51 (1.20, 1.90)	1.42 (1.14, 1.77)	
Active	0.86 (0.62, 1.20)	1.00 (ref.)	1.19 (0.83, 1.72)	1.18 (0.81, 1.71)	
**HbA1c**					.84
<7.0%	1.05 (0.84, 1.32)	1.00 (ref.)	1.17 (0.86, 1.59)	1.27 (0.94, 1.70)	
≥7.0%	1.18 (0.90, 1.55)	1.00 (ref.)	1.48 (1.09, 2.02)	1.27 (0.92, 1.76)	
**Hypertension**					.29
Yes	0.95 (0.71, 1.28)	1.00 (ref.)	1.10 (0.80, 1.52)	1.19 (0.85, 1.67)	
No	1.21 (0.86, 1.70)	1.00 (ref.)	1.10 (0.76, 1.59)	1.53 (1.13, 2.09)	
**Hypercholesterolemia**					.16
Yes	2.10 (1.12, 3.91)	1.00 (ref.)	2.04 (1.06, 3.93)	2.45 (1.35, 4.44)	
No	1.50 (0.90, 2.52)	1.00 (ref.)	1.57 (0.87, 2.83)	2.51 (1.47, 4.29)	

HRs (95% CIs) were adjusted for age (continuous), sex (male, or female), race/ethnicity (non-Hispanic white, non-Hispanic black, Mexican American, or other), BMI (<25.0, 25.0-29.9, or ≥30.0 kg/m^2), education level (less than high school, high school or equivalent, or college or above), family income level (lower, normal, higher), smoking status (never smoker, ever smoker, or current smoker), drinking status (never drinker, ever drinker, or current drinker), physical activity (inactive, or active), HEI (continuous), duration of diabetes (<3, 3-10, or >10 years), diabetes medication use (none, only diabetes pills, only insulin, or diabetes pill and insulin), HbA1c (<7%, ≥7%), self-reported hypertension (yes or no), and self-reported hypercholesterolemia (yes or no). The strata variable was not included when stratifying by itself.

© 2022 Liu Y et al. *JAMA Network Open*.

eTable 3. HRs (95% CIs) of CVD Mortality According to Serum Folate and Vitamin B_{12} Levels After Excluding T2D Patients With Less Than 2 Years of Follow-up

	**Serum folate concentrations (nmol/L)**				**Serum vitamin B_{12} concentrations (pmol/L)**			
	**Q1: <16.2**	**Q2: 16.2-27.4**	**Q3: 27.5-44.0**	**Q4: ≥44.1**	**Q1: <272.3**	**Q2: 272.3-373.3**	**Q3: 373.4-519.0**	**Q4: ≥519.1**
**CVD mortality**								
No. deaths/total	306/1802	149/1737	138/1730	90/1656	114/1064	72/1069	96/1061	104/1032
Model 1[a]	1.47 (1.00, 2.17)	1.00 (ref.)	1.31 (0.94, 1.83)	1.12 (0.79, 1.58)	1.85 (1.13, 3.00)	1.00 (ref.)	1.69 (1.07, 2.65)	2.88 (1.88, 4.41)
Model 2[b]	1.48 (1.03, 2.12)	1.00 (ref.)	1.44 (1.05, 1.98)	1.21 (0.86, 1.71)	1.89 (1.23, 2.91)	1.00 (ref.)	1.96 (1.27, 3.02)	2.78 (1.84, 4.20)

a Adjusted for age (continuous), sex (male, or female), and race (non-Hispanic white, non-Hispanic black, Mexican American, or other).

b Model 1+ BMI (<25.0, 25.0-29.9, or ≥30.0 kg/m^2), education level (less than high school, high school or equivalent, or college or above), family income level (lower, normal, higher), smoking status (never smoker, ever smoker, or current smoker), drinking status (never drinker, ever drinker, or current drinker), physical activity (inactive, active), HEI (continuous), duration of diabetes (<3, 3-10, or >10 years), diabetes medication use (none, only diabetes pills, only insulin, or diabetes pill and insulin), HbA1c (<7%, ≥7%), self-reported hypertension (yes, no), and self-reported hypercholesterolemia (yes, no).

© 2022 Liu Y et al. *JAMA Network Open*.

eTable 4. HR (95% CIs) of CVD Mortality According to Quintiles of Serum Folate and Vitamin B_{12} Concentrations Among Patients With T2D

	Serum folate concentrations (nmol/L)					Serum B_{12} concentrations (pmol/L)				
	**Q1 (<13.9)**	**Q2 (13.9-22.7)**	**Q3 (22.8-33.0)**	**Q4 (33.1-49.9)**	**Q5 (≥50.0)**	**Q1 (<253)**	**Q2 (253-332)**	**Q3 (333-421)**	**Q4 (422-564)**	**Q5 (≥565)**
**CVD mortality**										
No. deaths/total	302/1544	164/1536	122/1537	117/1545	94/1538	115/974	74/970	94/974	82/970	102/972
Model 1[a]	1.41 (1.07, 1.85)	1.00 (ref.)	0.85 (0.61, 1.18)	0.97 (0.71, 1.33)	0.86 (0.63, 1.16)	2.14 (1.33, 3.44)	1.00 (ref.)	1.84 (1.22, 2.78)	1.43 (0.97, 2.11)	2.88 (1.91, 4.36)
Model 2[b]	1.46 (1.09, 1.96)	1.00 (ref.)	0.90 (0.63, 1.29)	1.06 (0.77, 1.45)	0.96 (0.70, 1.33)	2.05 (1.32, 3.19)	1.00 (ref.)	1.85 (1.24, 2.76)	1.49 (1.01, 2.21)	2.66 (1.75, 4.04)

a Adjusted for age (continuous), sex (male, or female), and race (non-Hispanic white, non-Hispanic black, Mexican American, or other).

b Model 1+ BMI (<25.0, 25.0-29.9, or ≥30.0 kg/m^2), education level (less than high school, high school or equivalent, or college or above), family income level (lower, normal, higher), smoking status (never smoker, ever smoker, or current smoker), drinking status (never drinker, ever drinker, or current drinker), physical activity (inactive, or active), HEI (continuous), duration of diabetes (≤3, 3-10, or >10 years), diabetes medication use (none, only diabetes pills, only insulin, or diabetes pill and insulin), HbA1c (<7%, ≥7%), self-reported hypertension (yes or no), self-reported hypercholesterolemia (yes or no).

© 2022 Liu Y et al. *JAMA Network Open*.

eTable 5. HRs (95% CIs) of CVD Mortality According to Serum Folate and Vitamin B_{12} Levels Among T2D Patients With Further Adjustment of Several Biomarkers and Dietary Factors

		Serum folate concentrations (nmol/L)					Serum B_{12} concentrations (pmol/L)			
	N	Q1: <16.2	Q2: 16.2-27.4	Q3: 27.5-44.0	Q4: ≥44.1	N	Q1: <272.3	Q2: 272.3-373.3	Q3: 373.4-519.0	Q4: ≥519.1
No. deaths/total		345/1929	180/1921	159/1925	115/1925		138/1213	88/1216	117/1216	124/1215
Model 1	7700	1.43 (1.04, 1.98)	1.00 (ref.)	1.17 (0.88, 1.57)	1.03 (0.74, 1.44)	4860	1.74 (1.20, 2.52)	1.00 (ref.)	1.79 (1.19, 2.71)	2.32 (1.60, 3.35)
Model 2	4841	1.72 (1.03, 2.87)	1.00 (ref.)	1.27 (0.85, 1.89)	1.01 (0.62, 1.65)	4841	1.69 (1.15, 2.47)	1.00 (ref.)	1.80 (1.18, 2.74)	2.44 (1.61, 3.72)
Model 3	5480	1.53 (1.00, 2.34)	1.00 (ref.)	1.22 (0.82, 1.81)	1.20 (0.76, 1.91)	3481	1.54 (0.93, 2.56)	1.00 (ref.)	1.85 (1.04, 3.29)	2.71 (1.60, 4.62)
Model 4	6051	1.54 (0.92, 2.61)	1.00 (ref.)	1.24 (0.79, 1.92)	1.26 (0.80, 1.98)	3243	1.98 (1.04, 3.79)	1.00 (ref.)	2.08 (1.15, 3.76)	3.31 (1.89, 5.78)
Model 5	2849	1.40 (1.01, 1.94)	1.00 (ref.)	1.23 (0.91, 1.67)	1.11 (0.78, 1.59)	1842	1.63 (1.10, 2.43)	1.00 (ref.)	1.69 (1.11, 2.58)	2.22 (1.49, 3.30)
Model 6	3116	1.34 (0.76, 2.37)	1.00 (ref.)	1.34 (0.89, 2.01)	1.05 (0.62, 1.78)	3109	1.54 (1.00, 2.37)	1.00 (ref.)	1.74 (1.12, 2.71)	2.44 (1.63, 3.65)
Model 7	7066	1.41 (0.93, 2.04)	1.00 (ref.)	1.37 (0.95, 1.98)	1.16 (0.73, 1.83)	4825	1.67 (1.12, 2.50)	1.00 (ref.)	1.78 (1.18, 2.70)	2.25 (1.52, 3.33)
Model 8	7590	1.38 (0.97, 1.95)	1.00 (ref.)	1.30 (0.97, 1.74)	1.15 (0.80, 1.65)	4831	1.61 (1.05, 2.47)	1.00 (ref.)	1.56 (1.03, 2.38)	2.28 (1.53, 3.41)
Model 9	3671	1.56 (1.06, 2.12)	1.00 (ref.)	1.61 (1.10, 2.57)	1.34 (0.83, 2.17)	2378	1.98 (1.07, 3.66)	1.00 (ref.)	1.43 (0.73, 2.79)	3.05 (1.59, 5.83)

© 2022 Liu Y et al. *JAMA Network Open*.

Model 10	7700	1.53 (1.01, 2.30)	1.00 (ref.)	1.38 (0.94, 2.01)	1.14 (0.72, 1.79)	4860	1.71 (1.16, 2.51)	1.00 (ref.)	1.79 (1.19, 2.70)	2.27 (1.56, 3.32)

Model 1: Adjusted for age (continuous), sex (male, or female), and race (non-Hispanic white, non-Hispanic black, Mexican American, or other), BMI (<25.0, 25.0-29.9, or ≥30.0 kg/m^2), education level (less than high school, high school or equivalent, or college or above), family income level (lower, normal, higher), smoking status (never smoker, ever smoker, or current smoker), drinking status (never drinker, ever drinker, or current drinker), physical activity (inactive, active), HEI (continuous), duration of diabetes (<3, 3-10, or >10 years), diabetes medication use (none, only diabetes pills, only insulin, or diabetes pill and insulin), HbA1c (<7%, ≥7%), self-reported hypertension (yes, no), self-reported hypercholesterolemia (yes, no).

Model 2: Model 1 + mutually adjustment of serum folate and vitamin B12.

Model 3: further excluded participants who had CVD history at baseline based on Model 1.

Model 4: Model 1 + CRP.

Model 5: Model 1 + triglycerides, LDL, and HDL.

Model 6: Model 1 + homocysteine.

Model 7: Model 1 + AST, ALT, GGT, LDH.

Model 8: Model 1 + GFR.

Model 9: Model 1 + vitamin B12 supplements and folate supplements.

Model 10: Model 1 + dietary vitamin B12 and folate intake.

© 2022 Liu Y et al. *JAMA Network Open*.

eTable 6. HRs (95% CIs) of CVD Mortality According to Serum Vitamin B_{12} Levels Among Patients With T2D in NHANES III and NHANES 1999-2014

	**Serum vitamin B12 concentrations (pmol/L)**				
	**<272.3**	**272.3-373.3**	**373.4-519.0**	**519.1-701.0**	**≥701.1**
**CVD mortality**					
No. deaths/total	138/1213	88/1216	117/1216	78/708	46/507
Model 1[a]	1.80 (1.16, 2.78)	1.00 (ref.)	1.67 (1.08, 2.56)	2.63 (1.69, 4.10)	2.25 (1.45, 3.49)
Model 2[b]	1.73 (1.19, 2.53)	1.00 (ref.)	1.77 (1.17, 2.67)	2.63 (1.72, 4.05)	1.84 (1.14, 2.98)

[a] Adjusted for age (continuous), sex (male, or female), and race (non-Hispanic white, non-Hispanic black, Mexican American, or other).
[b] Model 1+ BMI (<25.0, 25.0-29.9, or ≥30.0 kg/m^2), education level (less than high school, high school or equivalent, or college or above), family income level (lower, normal, higher), smoking status (never smoker, ever smoker, or current smoker), drinking status (never drinker, ever drinker, or current drinker), physical activity (inactive, active), HEI (continuous), duration of diabetes (<3, 3-10, or >10 years), diabetes medication use (none, only diabetes pills, only insulin, or diabetes pill and insulin), HbA1c (<7%, ≥7%), self-reported hypertension (yes, no), and self-reported hypercholesterolemia (yes, no).

© 2022 Liu Y et al. *JAMA Network Open*.

eTable 7. Spearman Correlation of Baseline Serum Folate and B_{12} With Age, BMI, HbA1c, CRP, Triglycerides, LDL, HDL, and Homocysteine Among Patients With T2D

		Serum folate	Serum vitamin B12
**Age (years)**		0.18**	-0.02
	N	7700	4860
**BMI (kg/m^2)**		-0.02	-0.08**
	N	7700	4860
**HbA1c (%)**		0.01	0.07**
	N	7678	4852
**CRP (mg/dL)**		-0.15**	0.07**
	N	6051	3243
**Total cholesterol (mmol/L)**		-0.19**	0.02
	N	7649	4849
**Triglyceride (mmol/L)**		-0.02*	-0.06**
	N	7616	4838
**LDL (mmol/L)**		-0.17**	0.02
	N	2880	1853
**HDL (mmol/L)**		0.09**	0.09**
	N	7613	4831
**Homocysteine (umol/L)**		-0.09**	-0.22**
	N	3116	3109

*P<.05, **P<.01

© 2022 Liu Y et al. *JAMA Network Open*.

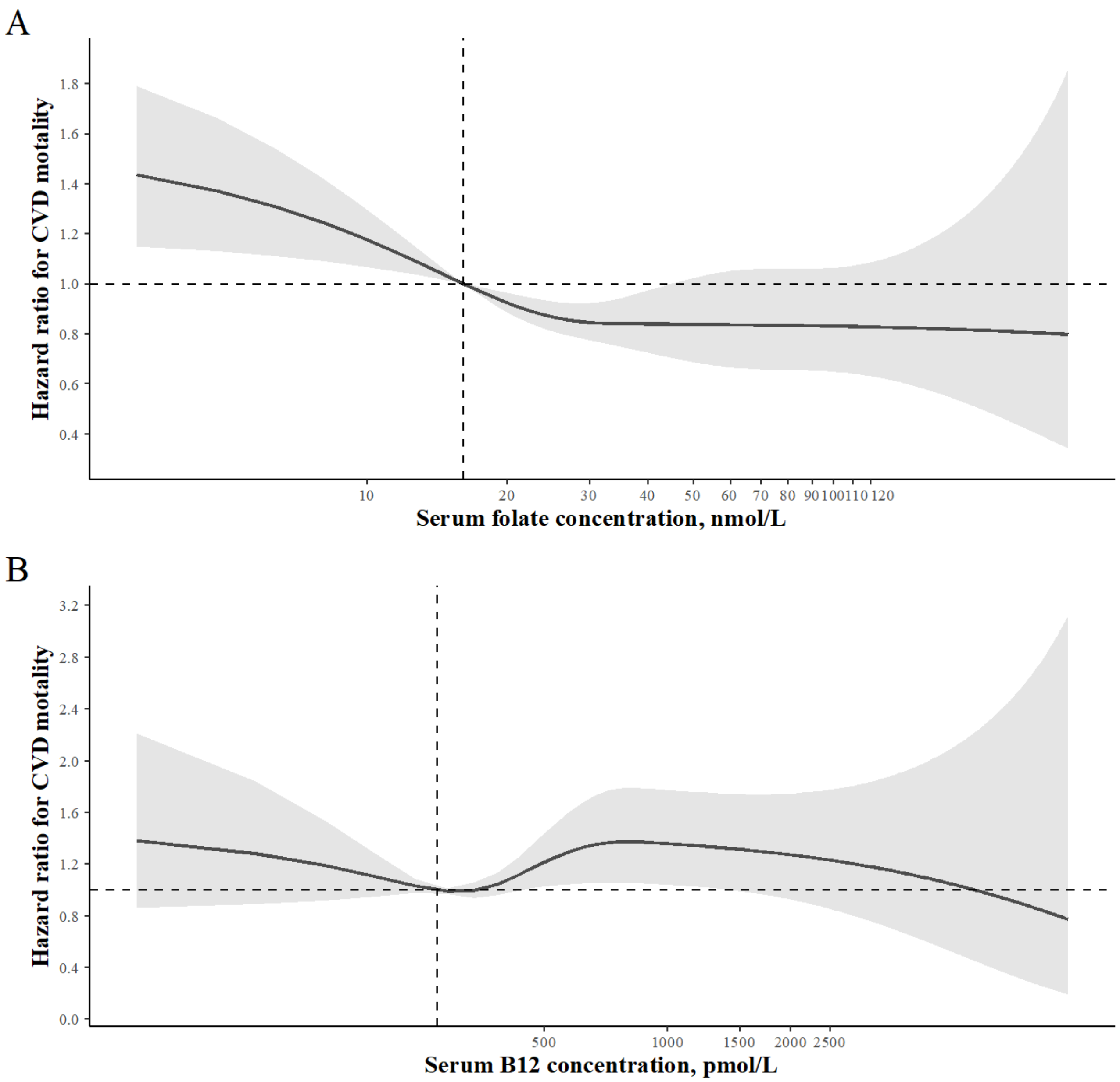

eFigure. Association of Serum Folate (A) and Vitamin B_{12} (B) With CVD Mortality Among Patients With T2D

HRs (95% CIs) were adjusted for age (continuous), sex (male, or female), race/ethnicity (non-Hispanic white, non-Hispanic black, Mexican American, or other), BMI (<25.0, 25.0-29.9, or ≥30.0 kg/m^2), education level (less than high school, high school or equivalent, or college or above), family income level (lower, normal, higher), smoking status (never smoker, ever smoker, or current smoker), drinking status (never drinker, ever drinker, or current drinker), physical activity (inactive, active), HEI (continuous), duration of diabetes (<3, 3-10, or >10 years), diabetes medication use (none, only diabetes pills, only insulin, or diabetes pill and insulin), HbA1c (<7%, ≥7%), self-reported hypertension (yes, no), and self-reported hypercholesterolemia (yes, no). P-nonlinearity=.02 in A. P-nonlinearity=.04 in B.

© 2022 Liu Y et al. *JAMA Network Open*.
